# Higher Risk of Lymph Node Metastasis in Young Patients with Early Gastric Cancer

**DOI:** 10.7150/jca.30260

**Published:** 2019-07-23

**Authors:** Zu-Kai Wang, Jian-Xian Lin, Ping Li, Jian-Wei Xie, Jia-Bin Wang, Jun Lu, Qi-Yue Chen, Long-Long Cao, Mi Lin, Ru-Hong Tu, Chang-Ming Huang, Chao-Hui Zheng

**Affiliations:** 1 Department of Gastric Surgery, Fujian Medical University Union Hospital, Fuzhou, Fujian Province, China; 2 Department of General Surgery, Fujian Medical University Union Hospital, Fuzhou, Fujian Province, China; 3 Key Laboratory of Ministry of Education of Gastrointestinal Cancer, Fujian Medical University, Fuzhou, Fujian Province, China; 4 Fujian Key Laboratory of Tumor Microbiology, Fujian Medical University, Fuzhou, Fujian Province, China

**Keywords:** lymph node metastasis, early gastric cancer, young, limited lymph node dissection

## Abstract

**Objective:** Whether age affects lymph node metastasis (LNM) in patients with gastric cancer (GC) is currently inconclusive. This study investigates the effect of age on LNM in patients with GC.

**Methods:** From January 1988 to December 2013, 22,808 GC patients underwent gastrectomy at the Surveillance, Epidemiology, and End Results database were included. The relationship between age and LNM was analyzed.

**Results:** The median number of examined lymph nodes (ELNs) was 12 (interquartile range [IQR], 7-20) among the 22,808 patients with GC, and the median numbers of ELNs were 10 (IQR, 5-18), 12 (IQR, 6-19), 13 (IQR, 7-21) and 13 (IQR, 7-21) in patients with T1 to T4 disease, respectively. A total of 13,780 (60.4%) patients presented with LNM. The LNM rates were 69.6%, 66.1%, 64.7%, 61.8%, 57.8% and 55.6% for patients in the 20-39, 40-49, 50-59, 60-69, 70-79 and ≥ 80 age groups, respectively (P < 0.001). The LNM rates and the number of positive lymph nodes were correlated with age among patients whose diseases were of the same T stage (all P < 0.01). Multivariate analysis showed that age was an independent predictor for LNM in patients with early gastric cancer (EGC) (P < 0.05), and linear regression analysis showed that the LNM rate was higher in young patients with EGC (P < 0.05).

**Conclusions:** Age is an independent predictor for LNM in EGC. Moreover, LNM is more common in young patients with EGC than in other age groups, which indicates that limited lymph node dissection may not be appropriate for young patients with EGC.

## Introduction

Gastric cancer (GC) is one of the most common malignant tumors of the digestive system and has had a significant impact on public health. GC is the 4^th^ most common malignant tumor and the 3^rd^ leading cause of cancer-related mortality 1. Lymph node metastasis (LNM) has an important effect on the prognosis and the extent of lymph node dissection of GC 2-4. However, whether age has an impact on LNM has not yet been conclusively determined. Nelen et al. analyzed the clinicopathological characteristics of patients with GC who were under and over 70 years of age and concluded that age was closely related to LNM 5. However, a study by Liu et al. involving 198 patients with GC who were under 40 years of age and 1,096 patients with GC who were over 55 years of age found that there was no significant difference in N stage between different age groups 6. In patients with early gastric cancer (EGC) who may receive limited lymph node dissection (D1 or D1+ lymph node dissection), there are no reports about whether LNM rates differ between different age groups. Therefore, the current study incorporated large sample data from the Surveillance, Epidemiology, and End Results (SEER) database to investigate the effect of age on LNM in patients with GC.

## Patients and Methods

### Patients

**Figure 1:**
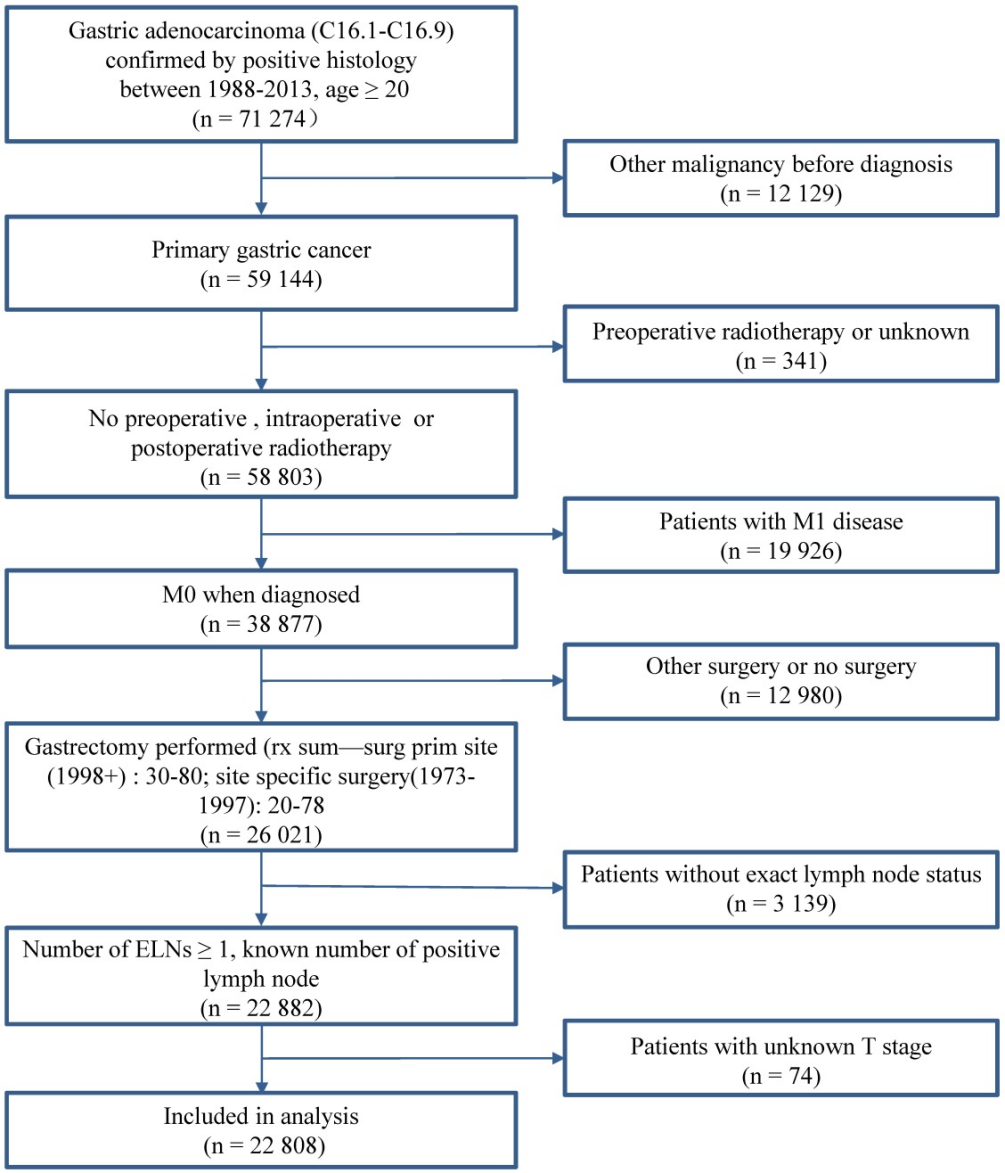
Case Screening Process. Abbreviation: ELNs, examined lymph nodes.

A total of 22,808 patients with GC who underwent gastrectomy between January 1988 and December 2013 in the SEER database (Nov 2015) were eligible for this study. The inclusion criteria were as follows: (1) histological determined GC (ICD-0-3M-8140/3, M-8142/3 through M-8145/3, M-8210/3, M-8211/3, M-8255/3, M-8260/3 through M-8263/3, M-8310/3, M-8323/3, M-8480/3, M-8481/3, and M-8490/3); (2) no record of previous malignant tumors before being diagnosed with GC; (3) number of examined nodes (ELNs) > 1; and (4) with complete clinicopathological information. The following patients were excluded from the study: (1) patients with distant metastasis (M1); (2) patients who received preoperative radiotherapy; and (3) patients with cardia carcinoma. Fig. 1 shows the stepwise process through which patients were selected for the study. The informed consent and institutional review board approval are not required in the study because the SEER database is open access without identification for all patients.

### Variables and Definitions

The following variables were included in the study: age, race, gender, depth of invasion, surgery type, tumor size, histological type, number of ELNs and the number of positive lymph nodes. Age was divided into 20-39 (defined as young), 40-49, 50-59, 60-69, 70-79 and ≥ 80 groups. Race included white, black and other. Postoperative pathological stage was defined according to the seventh edition of the AJCC/UICC stage standard. Surgery type analyzed in the study included partial gastrectomy (distal or proximal gastrectomy), near total/total gastrectomy, gastrectomy not otherwise specified (NOS) and combined resection according to SEER's surgical information. Cases were divided into groups of ≤ 45 mm and > 45 mm according to the median tumor size. The histological types in this study were classified into intestinal type (papillary adenocarcinoma and tubular adenocarcinoma) and diffuse type (carcinoma diffuse type, signet ring cell carcinoma and linitis plastica) according to the Lauren classification. We use “International Classification of Diseases for Oncology, 3rd Edition” to identify the histological type in SEER database 7. And the number of ELNs was divided into groups of ≤ 15 and > 15 according to National Comprehensive Cancer Network (NCCN) standards 8.

### Statistical Analysis

Chi-square tests were used to analyze the relationships between age and LMN rates in each T stage. The Jonckheere-Terpstra test was used to analyze the relationship between age and the number of positive lymph nodes in patients with LNM. Binary logistic regression analysis was used to analyze the risk factors for LNM and the covariates included age, gender, race, surgery type, tumor size, histological type and the number of ELNs. Linear regression was used to analyze the relationships between LNM rates and age. Statistical analyses were performed using SPSS 22.0 (SPSS Inc., Chicago, IL, USA). All statistical tests were 2-sided, and a P value < 0.05 was considered statistically significant.

## Results

### Patient Demographics and Tumor Characteristics

A total of 22,808 patients were enrolled in this study. Overall, 761 (3.3%) patients were under 40 years of age, 1,868 (8.2%) patients were between 40 and 49 years of age, 3,375 (14.8%) patients were between 50 and 59 years of age, 5,429 (23.8%) patients were between 60 and 69 years of age, 6,990 (30.6%) patients were between 70 and 79 years of age, and 4,385 (19.2%) patients were older than 80 years of age. A total of 4,849 (21.3%) patients had T1 disease, 2,735 (12%) patients had T2 disease, 8,267 (36.2%) patients had T3 disease, and 6,957 (30.5%) patients had T4 disease. A total of 9,028 (39.6%) patients had N0 stage, 4,163 (18.3%) patients had N1 stage, 4,254 (18.7%) patients had N2 stage, and 5,360 (23.5%) patients had N3 stage. Patients in different age groups differed significantly with respect to race, gender, T stage, N stage, surgery type, tumor size and histological type (Table 1).

### Lymph Node Dissection in Each Age Group

Overall, the median number of ELNs was 12 (interquartile range [IQR], 7-20). The median numbers of ELNs were 14 (IQR, 9-23), 15 (IQR, 8-23), 15 (IQR, 7-21), 13 (IQR, 7-21), 12 (IQR, 6-19) and 10 (IQR, 5-16.5) for patients in the 20-39, 40-49, 50-59, 60-69, 70-79 and ≥ 80 age groups, respectively (P < 0.001).

The median numbers of ELNs were 10 (IQR, 5-18), 12 (IQR, 6-19), 13 (IQR, 7-21) and 13 (IQR, 7-21) in patients with T1, T2, T3 and T4 disease, respectively. The median numbers of ELNs differed significantly between patients with different age groups whose diseases were of the same T stage (all P < 0.001) (Table 2).

### Association between Age and LNM

A total of 13,780 (60.4%) patients presented with LNM. The LNM rates were 69.6%, 66.1%, 64.7%, 61.8%, 57.8% and 55.6% for patients in the 20-39, 40-49, 50-59, 60-69, 70-79 and ≥ 80 age groups, respectively (P < 0.001). When stratified by depth of invasion, the LNM rates and the median number of positive lymph nodes were correlated with age in patients whose diseases were of the same T stage (all P < 0.01) (Table 3 and Table 4).

### Univariate and Multivariate Analysis of LNM

Univariate analysis showed that age was associated with LNM in the entire group and in each T stage (Table S1 to Table S5). Multivariate analysis showed that age, race, surgical type, tumor size, histological type, T stage and number of ELNs were independent predictors of LNM in the entire group (Table S1). Age was also an independent predictor for LNM in patients with T1 disease (P = 0.031), but was not an independent predictor for LNM in patients with T2-T4 disease (Table 5, Table S2 to Table S5).

### Linear Regression Analysis of Age and LNM Rates in Patients with EGC

**Figure 2:**
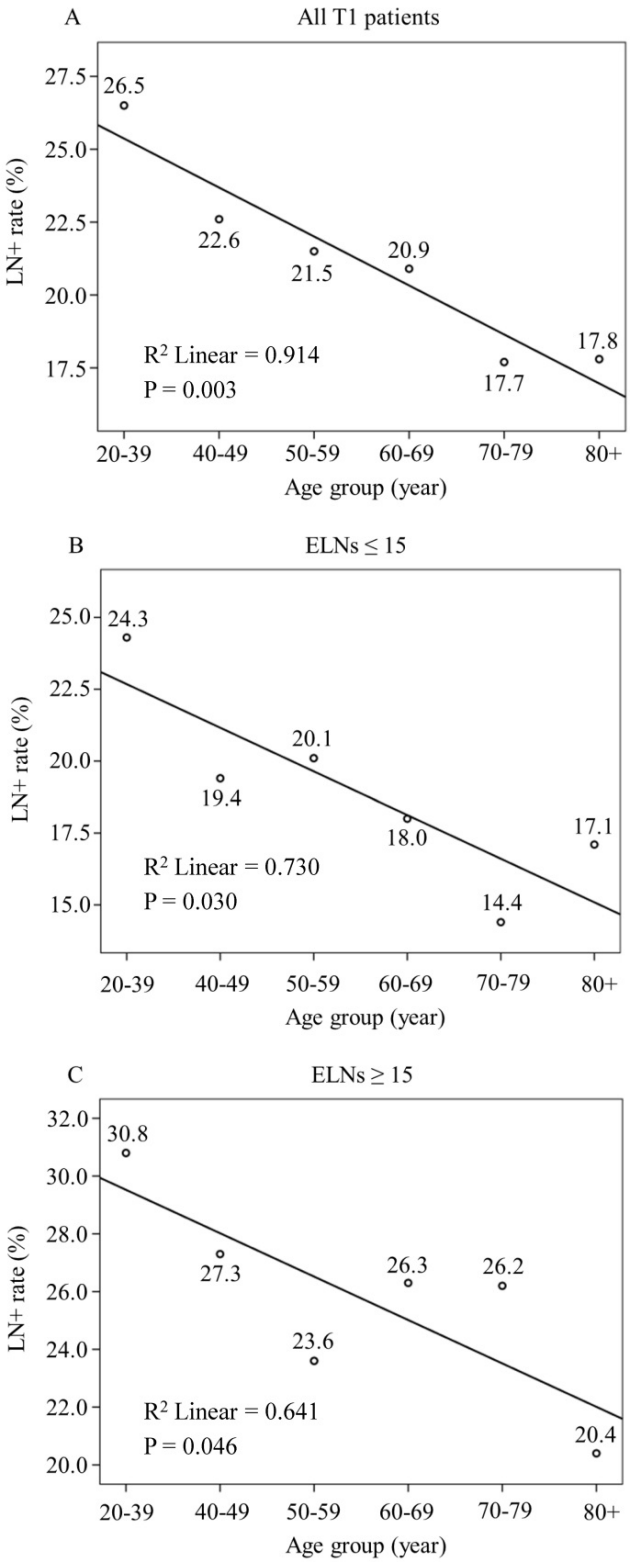
Linear Regression Analysis of the Relationship between Age and LNM Rates in Patients with EGC. LNM rate show a decrease trend with age increasing in (A) All patients with T1 disease (R^2^ Linear = 0.914, P = 0.003), (B) T1 patients with number of ELNs ≤ 15 (R^2^ Linear = 0.730, P = 0.030) and (C) T1 patients with > 15 ELNs (R^2^ Linear = 0.641, P = 0.046)

The LNM rate was 19.6% among the 4,849 patients with EGC. A total of 3,360 (69.3%) patients had ≤ 15 ELNs, and 1,489 (30.7%) patients had > 15 ELNs. The LNM rates were 17.1% and 25.3% in the > 15 and the ≤ 15 ELNs groups, respectively. Linear regression analysis showed that young patients with EGC are at higher risk for LNM than other age patients (all patients with T1 disease, P = 0.003; ELNs ≤ 15, P = 0.030 and ELNs > 15, P = 0.046) (Fig. 2).

## Discussion

Worldwide, GC is still one of the major malignant tumors that affect human health. LNM is the most common pattern of metastasis in GC and is an important factor for determining the treatment options and predicting its prognosis. Previous study showed that the LNM rates of EGC range from 8.4% to 20.1% 9, while the LNM rate of advanced GC is as high as 70% 10. Bruno et al. reported that the biological characteristics of node-negative GC are mild with good prognosis. In contrast, node-positive GC has a poor prognosis 3. Wu et al. analyzed the prognostic data of 510 patients with GC and found that the 5-year survival rates for patients without LNM and with 1-4 positive lymph nodes were 89.5% and 62.8%, respectively, with significant difference 4.

Many scholars have systematically investigated the risk factors for LNM in GC. These studies found that the depth of invasion, histological type, tumor size, macroscopic type and lymphovascular invasion were closely related to LNM 11-14. However, whether age affects LNM is still controversial. Pisanu et al. found no significant difference in N stage between patients with GC who were over and under 50 years of age 15. However, Smith et al. compared the clinicopathological data of 30 patients under 35 years of age with that of 320 patients over 35 years of age and found that the LNM rates of the two age groups were significantly different 16. However, these studies incorporated small sample data and analyzed age only as a binary categorical variable; thus, these results need to be validated in future studies. In the current study, a large number of cases with number of ELNs ≥ 1 were retrospectively analyzed, and age was divided into continuous intervals so that its relationship with LNM could be elucidated. For the entire group, univariate analysis found that age was closely related to LNM, and multivariate analysis showed that age was an independent predictor of LNM (P < 0.001, Table S1). Additionally, in the current study, we stratified analyzed the impact of age on LNM by different T stage. The results indicated that age is associated with LNM in patients with GC. We also recalculated patients with a number of ELNs > 15 for analysis. Univariate analysis showed that age, race, surgery type, tumor size, histological type, and T stage were associated with LNM. Multivariate analysis founded that tumor size, histological type, and T stage were independent predictors of LNM. Although the effect of age on LNM was not statistically significant (P = 0.063), it was observed that the odds ratios of other age groups were all less than 1 compared to young patients (Table S6), which indicating a higher risk of LNM in young patients. Gurzu et al. found that VEGF, a gene increases the likelihood of tumor invasion and LMN in GC, is overexpressed in younger patients with GC 17. Bao et al. argued that MDM4, which is related to LNM and a poor prognosis in GC, is overexpressed in younger patients compared with older patients 18. Based on the abovementioned studies and the results of our study, we hypothesized that age is closely associated with LNM in GC.

Some studies have shown that patients with EGC who underwent limited lymph node dissection can obtain a similar long-term survival compared with patients who underwent D2 radical surgery and the quality of life after surgery is significantly improved 19-23. According to "Japanese Gastric Cancer Treatment Guidelines", limited lymph node dissection is feasible for clinical node-negative EGC 24. But for EGC patients with LNM, limited lymph node dissection carries a high risk of postoperative recurrence 25, 26. However, there is no accurate way to identify LNM in EGC. Preoperative CT and endoscopic ultrasonography can only diagnose LMN with accuracies of 50% and 70%, respectively 27. Therefore, identifying the risk factors for LNM in EGC is important. Similar to previous studies, our study found that tumor size and histological type were independent predictive factors for LNM in patients with EGC 28, 29. However, multivariate analysis showed that age is an independent predictor for LNM in EGC (compared with young patients, the odds ratios for LNM in the other age groups ranged from 0.59 to 0.77). A linear regression analysis showed that the LNM rate was higher in young patients with EGC than in other age patients. EGC is a special type of GC which is located only in the mucosa and submucosa. Because lymphatic or blood vessels are less in the mucosa and submucosa, the risk factors of LNM in EGC are quite different from advanced GC. In this study, we found that age was an independent predictor for LNM in patients with T1 disease, but was not an independent predictor for LNM in patients with T2-T4 disease. The higher LNM rate in young patients with EGC compared to other age patients may be related to higher malignant potential of tumor in young patients. In the study conducted by Park et al., age was closely related to the tumor malignancy, and GC in younger patients was more aggressive than older patients 30. Based on previous study and findings of our study, we conclude that age has an important effect on LNM and the treatment options for EGC.

To our knowledge, this study for the first time investigates the relationship between age and LNM in patients with GC. We found that age is an independent predictor for LNM in patients with EGC and that LNM is more common in young patients with EGC than in other age patients. Therefore, clinicians should pay more attention to the preoperative assessment of lymph node status in young patients with EGC and realize that limited lymph node dissection may not be appropriate for these patients.

Our study had limitations that must be considered. First, this study had a retrospective design, which may have led to bias in the data selection process. Second, the information of neoadjuvant chemotherapy is not available in the SEER database, which may have a certain impact on LNM. Third, since the SEER database is a multi-center database, the quality of surgery may vary widely between centers. Nevertheless, we determined the impact of age on LNM in patients with EGC. These findings may serve as a basis for prospective studies and may ultimately affect the clinical treatment strategies for young patients with EGC.

## Supplementary Material

Supplementary tables.

## Figures and Tables

**Table 1:** Clinicopathological characteristics of each age group

			Age at diagnosis						P*
			20-39	40-49	50-59	60-69	70-79	80+	
Characteristic	No.	(%)	Percentage						
Age at diagnosis									
20-39	761	(3.3)							
40-49	1868	(8.2)							
50-59	3375	(14.8)							
60-69	5429	(23.8)							
70-79	6990	(30.6)							
80+	4385	(19.2)							
Race									< 0.001
White	13337	(58.5)	56.0	51.9	52.9	56.2	60.1	66.2	
Black	3433	(15.1)	17.9	21.4	19.2	16.0	13.0	10.7	
Other	6038	(26.5)	26.1	26.7	27.9	27.8	26.8	23.1	
Gender									< 0.001
Male	12853	(56.4)	48.4	55.5	61.3	61.5	56.9	47.1	
Female	9955	(43.6)	51.6	44.5	38.7	38.5	43.1	52.9	
T stage									< 0.001
T1	4849	(21.3)	14.8	18.2	19.9	21.8	22.9	21.4	
T2	2735	(12.0)	10.1	10.5	11.4	11.7	12.1	13.7	
T3	8267	(36.2)	35.0	37.5	35.6	36.6	35.8	36.7	
T4	6957	(30.5)	40.1	33.8	33.2	29.9	29.2	28.3	
N stage									< 0.001
N0	9028	(39.6)	30.4	33.9	35.3	38.2	42.2	44.4	
N1	4163	(18.3)	15.6	17.7	16.7	18.0	18.5	20.0	
N2	4254	(18.7)	23.3	18.8	19.3	18.9	18.1	17.9	
N3	5363	(23.5)	30.7	29.6	28.6	24.9	21.2	17.7	
Surgery type									< 0.001
Partial gastrectomy	15556	(68.2)	58.2	60.0	62.4	64.9	70.4	78.5	
Near total/total gastrectomy	4053	(17.8)	22.5	22.7	21.2	20.1	16.2	11.8	
Gastrectomy NOS	297	(1.3)	1.6	1.6	1.8	1.2	1.3	0.9	
Combined resection	2902	(12.7)	17.7	15.7	14.6	13.8	12.1	8.8	
Tumor size									< 0.001
≤ 45mm	10092	(44.2)	40.5	41.8	42.4	45.0	44.9	45.3	
> 45mm	9792	(42.9)	44.8	43.5	42.8	41.9	42.6	44.2	
Unknown	2924	(12.8)	14.7	14.7	14.8	13.1	12.5	10.4	
Histological type									< 0.001
Intestinal	21038	(92.2)	85.7	87.9	90.0	91.7	93.8	95.2	
Diffuse	1770	(7.8)	14.3	12.1	10.0	8.3	6.2	4.8	

*P value from chi-square test Due to rounding, the values in parentheses may not add up to 100 **Abbreviations:** NOS, not otherwise specified

**Table 2:** Number of ELNs in each age group by T stage

Age	N	Median	25^th^, 75^th^		P*
Group, y		ELNs	Percentile		
All T stage					< 0.001
Total	22808	12	7	20	
20-39	761	14	9	23	
40-49	1868	15	8	23	
50-59	3375	15	8	23	
60-69	5429	13	7	21	
70-79	6990	12	6	19	
80+	4385	10	5	16.5	
T stage T1					< 0.001
Total	4849	10	5	18	
20-39	113	12	6	19.5	
40-49	340	13	6	22	
50-59	671	12	6	20	
60-69	1185	11	6	19	
70-79	1603	9	5	16	
80+	937	8	4	14	
T stage T2					< 0.001
Total	2735	12	6	19	
20-39	77	15	9	22	
40-49	196	13.5	7.25	22	
50-59	385	14	8	23	
60-69	635	13	7	20	
70-79	843	11	7	18	
80+	599	9	5	15	
T stage T3					< 0.001
Total	8267	13	7	21	
20-39	266	14	9	21.3	
40-49	701	15	9	22	
50-59	1200	15	9	24	
60-69	1988	14	8	22	
70-79	2503	12	7	20	
80+	1609	10	6	17	
T stage T4					< 0.001
Total	6957	13	7	21	
20-39	305	15	8	24.5	
40-49	631	15	9	23	
50-59	1119	15	9	23	
60-69	1621	14	8	22	
70-79	2041	12	7	20	
80+	1240	11	6	18	

*P value from Jonckheere-Terpstra test

**Table 3:** LNM rates in each age group by T stage

Age	N	LN positive		P*
Group, y		No.	(%)	
All T stage				< 0.001
Total	22808	13780	60.4	
20-39	761	530	69.6	
40-49	1868	1235	66.1	
50-59	3375	2182	64.7	
60-69	5429	3356	61.8	
70-79	6990	4040	57.8	
80+	4385	2437	55.6	
T stage T1				0.001
Total	4849	949	19.6	
20-39	113	30	26.5	
40-49	340	77	22.6	
50-59	671	144	21.5	
60-69	1185	248	20.9	
70-79	1603	283	17.7	
80+	937	167	17.8	
T stage T2				0.001
Total	2735	1237	45.2	
20-39	77	42	54.5	
40-49	196	98	50	
50-59	385	198	51.4	
60-69	635	281	44.3	
70-79	843	361	42.8	
80+	599	257	42.9	
T stage T3				< 0.001
Total	8267	5913	71.5	
20-39	266	200	75.2	
40-49	701	526	75	
50-59	1200	913	76.1	
60-69	1988	1481	74.5	
70-79	2503	1749	69.9	
80+	1609	1044	64.9	
T stage T4				< 0.001
Total	6957	5681	81.7	
20-39	305	258	84.6	
40-49	631	534	84.6	
50-59	1119	927	82.8	
60-69	1621	1346	83	
70-79	2041	1647	80.7	
80+	1240	969	78.1	

*P value from chi-square test

**Table 5:** Uni- and multivariate binary logistic regression analysis of the relationship between age and LNM in each T stage

Age	Univariate Analysis					Multivariate Analysis			
group, y	Odds Ratio	95%CI		P		Odds Ratio	95%CI		P
All T stage				<0.001					<0.001
20-39	Ref					Ref			
40-49	0.85	0.71	1.02	0.081		0.92	0.75	1.13	0.435
50-59	0.80	0.67	0.95	0.009		0.91	0.75	1.11	0.349
60-69	0.71	0.60	0.83	<0.001		0.87	0.72	1.05	0.146
70-79	0.60	0.51	0.70	<0.001		0.76	0.63	0.91	0.003
80+	0.55	0.46	0.64	<0.001		0.70	0.58	0.85	<0.001
T stage T1				0.016					0.031
20-39	Ref					Ref			
40-49	0.81	0.50	1.32	0.398		0.74	0.45	1.23	0.247
50-59	0.76	0.48	1.19	0.230		0.77	0.48	1.22	0.259
60-69	0.73	0.47	1.14	0.165		0.72	0.46	1.13	0.153
70-79	0.59	0.38	0.92	0.019		0.59	0.38	0.93	0.022
80+	0.60	0.38	0.94	0.026		0.59	0.37	0.93	0.023
T stage T2				0.016					0.100
20-39	Ref					Ref			
40-49	0.83	0.49	1.41	0.499		0.83	0.49	1.41	0.488
50-59	0.88	0.54	1.44	0.617		0.85	0.52	1.40	0.524
60-69	0.66	0.41	1.06	0.088		0.66	0.41	1.07	0.093
70-79	0.62	0.39	1.00	0.049		0.65	0.40	1.04	0.073
80+	0.63	0.39	1.01	0.054		0.67	0.41	1.08	0.098
T stage T3				0.023					0.138
20-39	Ref					Ref			
40-49	0.99	0.72	1.38	0.961		1.00	0.72	1.40	0.984
50-59	1.05	0.77	1.43	0.757		1.04	0.76	1.42	0.798
60-69	0.96	0.72	1.30	0.808		1.01	0.75	1.36	0.960
70-79	0.77	0.57	1.02	0.072		0.83	0.62	1.12	0.217
80+	0.61	0.45	0.82	0.001		0.71	0.52	0.96	0.025
T stage T4				0.001					0.376
20-39	Ref					Ref			
40-49	1.00	0.69	1.47	0.988		1.00	0.68	1.47	0.991
50-59	0.88	0.62	1.25	0.469		0.89	0.63	1.27	0.523
60-69	0.89	0.64	1.25	0.505		0.94	0.67	1.32	0.710
70-79	0.76	0.55	1.06	0.105		0.85	0.61	1.19	0.353
80+	0.65	0.46	0.91	0.013		0.79	0.56	1.12	0.184

**Abbreviations:** NOS, not otherwise specified; Ref, reference; CI, confidence interval. Covariates included age, gender, race, surgery type, tumor size, histological type, and the number of ELNs.

**Table 4:** Association between age and the number of positive LNs in patients with LNM

Age	Median Positive LNs	25^th^, 75^th^		
group, y		Percentile		P*
All T stage				< 0.001
Total	5	2	10	
20-39	6	3	11	
40-49	6	2	11	
50-59	6	2	10	
60-69	5	2	10	
70-79	4	2	9	
80+	4	2	8	
T stage T1				0.002
Total	2	1	4	
20-39	2.5	1	6	
40-49	2	1	6	
50-59	2	1	4	
60-69	2	1	4	
70-79	2	1	4	
80+	1	1	3	
T stage T2				< 0.001
Total	3	1	5	
20-39	4	2	6	
40-49	3	1	5	
50-59	3	1	7	
60-69	3	1	5	
70-79	2	1	5	
80+	2	1	4	
T stage T3				< 0.001
Total	5	2	9	
20-39	6	3	11	
40-49	6	2	10	
50-59	6	3	10	
60-69	5	2	10	
70-79	4	2	9	
80+	4	2	8	
T stage T4				< 0.001
Total	6	3	12	
20-39	6	3	13	
40-49	7	3	12	
50-59	7	3	12	
60-69	6	3	12	
70-79	6	3	12	
80+	5	2	11	

*P value from Jonckheere-Terpstra test

## Abbreviations

LNM: lymph node metastasis
GC: gastric cancer
ELNs: examined lymph nodes
IQR: interquartile range
EGC: early gastric cancer
SEER: the Surveillance, Epidemiology, and End Results
NOS: not otherwise specified
NCCN: National Comprehensive Cancer Network
CI: confidence interval

## References

[1] Torre LA, Bray F, Siegel RL, Ferlay J, Lortet-Tieulent J, Jemal A (2015). Global cancer statistics, 2012. CA: a cancer journal for clinicians.

[2] Roukos DH (1998). Extended lymphadenectomy in gastric cancer: when, for whom and why. Annals of the Royal College of Surgeons of England.

[3] Bruno L, Nesi G, Montinaro F, Carassale G, Boddi V, Bechi P (2000). Clinicopathologic characteristics and outcome indicators in node-negative gastric cancer. Journal of surgical oncology.

[4] Wu CW, Hsieh MC, Lo SS, Tsay SH, Li AF, Lui WY (1997). Prognostic indicators for survival after curative resection for patients with carcinoma of the stomach. Digestive diseases and sciences.

[5] Nelen SD, Verhoeven RHA, Lemmens V, de Wilt JHW, Bosscha K (2017). Increasing survival gap between young and elderly gastric cancer patients. Gastric cancer: official journal of the International Gastric Cancer Association and the Japanese Gastric Cancer Association.

[6] Liu S, Feng F, Xu G, Liu Z, Tian Y, Guo M (2016). Clinicopathological features and prognosis of gastric cancer in young patients. BMC cancer.

[7] Fritz A, Percy C, Jack A, Shanmugarathan S, Sobin LH, Parkin DM (2000). International Classification of Diseases for Oncology. 3rd ed.

[8] Ajani JA, D'Amico TA, Almhanna K, Bentrem DJ, Chao J, Das P (2016). Gastric Cancer, Version 3.2016, NCCN Clinical Practice Guidelines in Oncology. Journal of the National Comprehensive Cancer Network: JNCCN.

[9] Pelz J, Merkel S, Horbach T, Papadopoulos T, Hohenberger W (2004). Determination of nodal status and treatment in early gastric cancer. European journal of surgical oncology: the journal of the European Society of Surgical Oncology and the British Association of Surgical Oncology.

[10] Kurihara M, Aiko T, Japanese Gastric Cancer A (2001). The new Japanese classification of gastric carcinoma: revised explanation of "response assessment of chemotherapy and radiotherapy for gastric carcinoma". Gastric cancer: official journal of the International Gastric Cancer Association and the Japanese Gastric Cancer Association.

[11] Chen S, Nie RC, OuYang LY, Li YF, Xiang J, Zhou ZW (2017). Nomogram analysis and external validation to predict the risk of lymph node metastasis in gastric cancer. Oncotarget.

[12] An JY, Baik YH, Choi MG, Noh JH, Sohn TS, Kim S (2007). Predictive factors for lymph node metastasis in early gastric cancer with submucosal invasion: analysis of a single institutional experience. Annals of surgery.

[13] Maehara Y, Orita H, Okuyama T, Moriguchi S, Tsujitani S, Korenaga D (1992). Predictors of lymph node metastasis in early gastric cancer. The British journal of surgery.

[14] Zhang Y, Zhu Z, Sun Z, Wang Z, Zheng X, Xu H (2014). Preoperative predicting score of lymph node metastasis for gastric cancer. Tumour biology: the journal of the International Society for Oncodevelopmental Biology and Medicine.

[15] Pisanu A, Podda M, Cois A, Uccheddu A (2014). Gastric cancer in the young: is it a different clinical entity? A retrospective cohort study. Gastroenterology research and practice.

[16] Smith BR, Stabile BE (2009). Extreme aggressiveness and lethality of gastric adenocarcinoma in the very young. Archives of surgery.

[17] Gurzu S, Kadar Z, Sugimura H, Bara T, Bara T Jr, Halmaciu I (2015). Gastric cancer in young vs old Romanian patients: immunoprofile with emphasis on maspin and mena protein reactivity. APMIS: acta pathologica, microbiologica, et immunologica Scandinavica.

[18] Bao J, Nanding A, Song H, Xu R, Qu G, Xue Y (2016). The overexpression of MDM4: an effective and novel predictor of gastric adenocarcinoma lymph node metastasis. Oncotarget.

[19] Shimoyama S, Seto Y, Yasuda H, Mafune K, Kaminishi M (2005). Concepts, rationale, and current outcomes of less invasive surgical strategies for early gastric cancer: data from a quarter-century of experience in a single institution. World journal of surgery.

[20] Memon MA, Subramanya MS, Khan S, Hossain MB, Osland E, Memon B (2011). Meta-analysis of D1 versus D2 gastrectomy for gastric adenocarcinoma. Annals of surgery.

[21] Yang SH, Zhang YC, Yang KH, Li YP, He XD, Tian JH (2009). An evidence-based medicine review of lymphadenectomy extent for gastric cancer. American journal of surgery.

[22] Yoshikawa T, Tsuburaya A, Kobayashi O, Sairenji M, Motohashi H, Noguchi Y (2002). Is D2 lymph node dissection necessary for early gastric cancer?. Annals of surgical oncology.

[23] Nomura S, Kaminishi M (2007). Surgical treatment of early gastric cancer. Digestive surgery.

[24] Japanese Gastric Cancer A (2017). Japanese gastric cancer treatment guidelines 2014 (ver. 4). Gastric cancer: official journal of the International Gastric Cancer Association and the Japanese Gastric Cancer Association.

[25] Jeon HK, Kim GH, Lee BE, Park DY, Song GA, Kim DH (2018). Long-term outcome of endoscopic submucosal dissection is comparable to that of surgery for early gastric cancer: a propensity-matched analysis. Gastric cancer: official journal of the International Gastric Cancer Association and the Japanese Gastric Cancer Association.

[26] Yano T, Ishido K, Tanabe S, Wada T, Azuma M, Kawanishi N (2018). Long-term outcomes of patients with early gastric cancer found to have lesions for which endoscopic treatment is not indicated on histopathological evaluation after endoscopic submucosal dissection. Surgical endoscopy.

[27] Park SR, Lee JS, Kim CG, Kim HK, Kook MC, Kim YW (2008). Endoscopic ultrasound and computed tomography in restaging and predicting prognosis after neoadjuvant chemotherapy in patients with locally advanced gastric cancer. Cancer.

[28] Kim YH, Park JH, Park CK, Kim JH, Lee SK, Lee YC (2017). Histologic purity of signet ring cell carcinoma is a favorable risk factor for lymph node metastasis in poorly cohesive, submucosa-invasive early gastric carcinoma. Gastric cancer: official journal of the International Gastric Cancer Association and the Japanese Gastric Cancer Association.

[29] Kim YI, Lee JH, Kook MC, Lee JY, Kim CG, Ryu KW (2016). Lymph node metastasis risk according to the depth of invasion in early gastric cancers confined to the mucosal layer. Gastric cancer: official journal of the International Gastric Cancer Association and the Japanese Gastric Cancer Association.

[30] Park JC, Lee YC, Kim JH, Kim YJ, Lee SK, Hyung WJ (2009). Clinicopathological aspects and prognostic value with respect to age: an analysis of 3,362 consecutive gastric cancer patients. Journal of surgical oncology.

