# Supplementary material for: Higher Risk of Lymph Node Metastasis in Young Patients with Early Gastric Cancer
Source: J Cancer. 2019 Jul 23;10(18):4389–96. doi: 10.7150/jca.30260 (PMC6691700; doi:10.7150/jca.30260)
Supplement: Supplementary file 1 — Supplementary tables. [file jcav10p4389s1.pdf]

Table S1. Uni- and multivariate binary logistic regression analysis for LNM in all patients

Parameter	Univariate Analysis				Multivariate Analysis			
	Odds Ratio	95%CI		P	Odds Ratio	95%CI		P
Age				<0.001				<0.001
20-39	Ref				Ref			
40-49	0.85	0.71	1.02	0.081	0.92	0.75	1.13	0.435
50-59	0.80	0.67	0.95	0.009	0.91	0.75	1.11	0.349
60-69	0.71	0.60	0.83	<0.001	0.87	0.72	1.05	0.146
70-79	0.60	0.51	0.70	<0.001	0.76	0.63	0.91	0.003
80+	0.55	0.46	0.64	<0.001	0.70	0.58	0.85	<0.001
Sex								
Female	Ref				Ref			
Male	1.06	1.00	1.12	0.039	1.03	0.97	1.09	0.384
Race				<0.001				0.026
Black	Ref				Ref			
White	0.95	0.88	1.02	0.176	0.89	0.81	0.97	0.008
Other	0.85	0.78	0.93	<0.001	0.93	0.84	1.09	0.135
Surgery type				<0.001				0.001
Partial gastrectomy	Ref				Ref			
Near total/total gastrctomy	1.70	1.57	1.82	<0.001	1.13	1.04	1.24	0.004
Gastrctomy NOS	1.60	1.25	2.03	<0.001	1.17	0.89	1.54	0.273
Combined resection	2.02	1.85	2.20	<0.001	1.18	1.07	1.31	0.001
Tumor size				<0.001				<0.001
≤45mm	Ref				Ref			
>45mm	3.06	2.89	3.25	<0.001	1.43	1.34	1.54	<0.001
Unknown	1.23	1.13	1.33	<0.001	1.05	0.95	1.16	0.308
Histological type								
Intestinal	Ref				Ref			
Diffuse	1.74	1.56	1.93	<0.001	1.26	1.11	1.42	<0.001
T stage				<0.001				<0.001
T1	Ref				Ref			
T2	3.40	3.06	3.76	<0.001	3.20	2.88	3.56	<0.001
T3	10.32	9.48	11.25	<0.001	8.83	8.10	9.66	<0.001
T4	18.30	16.67	20.10	<0.001	14.89	13.48	16.44	<0.001
Number of ELNs								
≤15	Ref				Ref			
>15	1.89	1.78	2.00	<0.001	1.67	1.57	1.79	<0.001

Abbreviations: NOS, not otherwise specified; Ref, reference; CI, confidence interval

Table S2. Uni- and multivariate binary logistic regression analysis for LNM in T1 patients

Parameter	Univariate Analysis				Multivariate Analysis			
	Odds Ratio	95%CI		P	Odds Ratio	95%CI		P
Age				0.016				0.031
20-39	Ref				Ref			
40-49	0,81	0.50	1.32	0.398	0.74	0.45	1.23	0.247
50-59	0.76	0.48	1.19	0.230	0.77	0.48	1.22	0.259
60-69	0.73	0.47	1.14	0.165	0.72	0.46	1.13	0.153
70-79	0.59	0.38	0.92	0.019	0.59	0.38	0.93	0.022
80+	0.60	0.38	0.94	0.026	0.59	0.37	0.93	0.023
Sex								
Female	Ref							
Male	0.87	0.75	1.00	0.053				
Race				0.034				0.018
Black	Ref				Ref			
White	0.89	0.73	1.09	0.264	0.91	0.74	1.13	0.397
Other	0.76	0.61	0.95	0.014	0.75	0.60	0.94	0.012
Surgery type				0.095				
Partial gastrectomy	Ref							
Near total/total gastrctomy	1.12	0.91	1.39	0.285				
Gastrctomy NOS	1.98	1.05	3.76	0.036				
Combined resection	1.18	0.90	1.56	0.239				
Tumor size				<0.001				<0.001
≤45mm	Ref				Ref			
>45mm	2.81	2.32	3.42	<0.001	2.74	2.25	3.34	<0.001
Unknown	0.69	0.56	0.85	<0.001	0.71	0.57	0.87	0.001
Histological type								
Intestinal	Ref				Ref			
Diffuse	1.44	1.08	1.94	0.015	1.29	0.95	1.76	0.101
Number of ELNs								
≤15	Ref				Ref			
>15	1.64	1.42	1.90	<0.001	1.50	1.29	1.75	<0.001

Abbreviations: NOS, not otherwise specified; Ref, reference; CI, confidence interval

Table S3. Uni- and multivariate binary logistic regression analysis for LNM in T2 patients

Parameter	Univariate Analysis				Multivariate Analysis			
	Odds Ratio	95%CI		P	Odds Ratio	95%CI		P
Age				0.016				0.100
20-39	Ref				Ref			
40-49	0.83	0.49	1.41	0.499	0.83	0.49	1.41	0.488
50-59	0.88	0.54	1.44	0.617	0.85	0.52	1.40	0.524
60-69	0.66	0.41	1.06	0.088	0.66	0.41	1.07	0.093
70-79	0.62	0.39	1.00	0.049	0.65	0.40	1.04	0.073
80+	0.63	0.39	1.01	0.054	0.67	0.41	1.08	0.098
Sex								
Female	Ref							
Male	0.89	0.76	1.04	0.131				
Race				0.087				
Black	Ref							
White	0.86	0.70	1.07	0.179				
Other	0.77	0.60	0.97	0.028				
Surgery type				0.663				
Partial gastrectomy	Ref							
Near total/total gastrctomy	1.00	0.80	1.24	0.972				
Gastrctomy NOS	1.32	0.62	2.83	0.470				
Combined resection	1.16	0.88	1.52	0.299				
Tumor size				0.001				0.004
≤45mm	Ref				Ref			
>45mm	1.36	1.15	1.61	<0.001	1.32	1.11	1.57	0.001
Unknown	0.96	0.73	1.26	0.769	0.97	0.74	1.27	0.815
Histological type								
Intestinal	Ref							
Diffuse	1.14	0.81	1.59	0.449				
Number of ELNs								
≤15	Ref				Ref			
>15	1.69	1.44	1.98	<0.001	1.62	1.38	1.90	<0.001

Abbreviations: NOS, not otherwise specified; Ref, reference; CI, confidence interval

Table S4. Uni- and multivariate binary logistic regression analysis for LNM in T3 patients

Parameter	Univariate Analysis				Multivariate Analysis			
	Odds Ratio	95%CI		P	Odds Ratio	95%CI		P
Age				0.023				0.138
20-39	Ref				Ref			
40-49	0.99	0.72	1.38	0.961	1.00	0.72	1.40	0.984
50-59	1.05	0.77	1.43	0.757	1.04	0.76	1.42	0.798
60-69	0.96	0.72	1.30	0.808	1.01	0.75	1.36	0.960
70-79	0.77	0.57	1.02	0.072	0.83	0.62	1.12	0.217
80+	0.61	0.45	0.82	0.001	0.71	0.52	0.96	0.025
Sex								
Female	Ref				Ref			
Male	1.14	1.03	1.25	0.010	1.11	1.00	1.22	0.043
Race				<0.001				<0.001
Black	Ref				Ref			
White	0.83	0.72	0.95	0.008	0.87	0.76	1.00	0.053
Other	1.15	0.98	1.36	0.082	1.14	0.96	1.34	0.128
Surgery type				<0.001				<0.001
Partial gastrectomy	Ref				Ref			
Near total/total gastrctomy	1.42	1.25	1.62	<0.001	1.22	1.07	1.39	0.003
Gastrctomy NOS	1.17	0.79	1.74	0.423	1.12	0.75	1.67	0.574
Combined resection	1.55	1.32	1.83	<0.001	1.39	1.18	1.65	<0.001
Tumor size				<0.001				<0.001
≤45mm	Ref				Ref			
>45mm	1.43	1.29	1.58	<0.001	1.35	1.22	1.50	<0.001
Unknown	1.24	1.05	1.47	0.011	1.24	1.04	1.47	0.016
Histological type								
Intestinal	Ref				Ref			
Diffuse	1.39	1.15	1.70	0.001	1.24	1.02	1.52	0.034
Number of ELNs								
≤15	Ref				Ref			
>15	1.67	1.51	1.85	<0.001	1.49	1.34	1.65	<0.001

Abbreviations: NOS, not otherwise specified; Ref, reference; CI, confidence interval

Table S5. Uni- and multivariate binary logistic regression analysis for LNM in T4 patients

Parameter	Univariate Analysis Odds Ratio	95%CI		P	Multivariate Analysis Odds Ratio	95%CI		P
Age				0.001				0.376
20-39	Ref				Ref			
40-49	1.00	0.69	1.47	0.988	1.00	0.68	1.47	0.991
50-59	0.88	0.62	1.25	0.469	0.89	0.63	1.27	0.523
60-69	0.89	0.64	1.25	0.505	0.94	0.67	1.32	0.710
70-79	0.76	0.55	1.06	0.105	0.85	0.61	1.19	0.353
80+	0.65	0.46	0.91	0.013	0.79	0.56	1.12	0.184
Sex								
Female	Ref							
Male	1.13	1.00	1.28	0.051				
Race				0.028				0.105
Black	Ref				Ref			
White	0.88	0.73	1.05	0.159	0.85	0.71	1.03	0.093
Other	1.06	0.87	1.31	0.559	0.97	0.79	1.20	0.778
Surgery type				<0.001				0.087
Partial gastrectomy	Ref				Ref			
Near total/total gastrctomy	1.53	1.30	1.81	<0.001	1.24	1.04	1.47	0.016
Gastrctomy NOS	1.01	0.62	1.66	0.968	0.85	0.51	1.41	0.531
Combined resection	1.29	1.10	1.52	0.002	1.08	0.91	1.27	0.373
Tumor size				<0.001				<0.001
≤45mm	Ref				Ref			
>45mm	1.49	1.30	1.71	<0.001	1.40	1.22	1.61	<0.001
Unknown	1.34	1.10	1.64		1.31	1.06	1.61	0.011
Histological type								
Intestinal	Ref				Ref			
Diffuse	1.51	1.22	1.88	<0.001	1.32	1.05	1.64	0.015
Number of ELNs								
≤15	Ref				Ref			
>15	2.35	2.05	2.69	<0.001	2.20	1.91	2.52	<0.001

Abbreviations: NOS, not otherwise specified; Ref, reference; CI, confidence interval

Table S6. Uni- and multivariate binary logistic regression analysis for LNM in all patients with ELNs > 15

Parameter	Univariate Analysis				Multivariate Analysis			
	Odds Ratio	95%CI		P	Odds Ratio	95%CI		P
Age				0.001				0.063
20-39	Ref				Ref			
40-49	0.73	0.55	0.98	0.037	0.82	0.59	1.14	0.236
50-59	0.75	0.57	0.98	0.036	0.88	0.64	1.20	0.407
60-69	0.67	0.52	0.88	0.004	0.84	0.62	1.14	0.269
70-79	0.64	0.49	0.84	0.001	0.79	0.58	1.06	0.117
80+	0.59	0.45	0.78	<0.001	0.68	0.50	0.94	0.018
Sex								
Female	Ref							
Male	1.04	0.95	1.14	0.450				
Race				0.001				0.580
Black	Ref				Ref			
White	1.04	0.91	1.19	0.550	0.95	0.82	1.12	0.583
Other	0.86	0.76	1.00	0.045	1.02	0.86	1.21	0.842
Surgery type				<0.001				0.185
Partial gastrectomy	Ref				Ref			
Near total/total gastrctomy	1.49	1.33	1.67	<0.001	1.08	0.95	1.23	0.266
Gastrctomy NOS	1.39	0.91	2.11	0.124	0.89	0.56	1.43	0.636
Combined resection	1.99	1.73	2.30	<0.001	1.18	1.00	1.39	0.046
Tumor size				<0.001				<0.001
≤45mm	Ref				Ref			
>45mm	2.93	2.65	3.24	<0.001	1.36	1.20	1.53	<0.001
Unknown	1.49	1.27	1.74	<0.001	1.03	0.86	1.24	0.756
Histological type								
Intestinal	Ref				Ref			
Diffuse	1.56	1.32	1.84	<0.001	1.32	1.08	1.60	0.006
T stage				<0.001				<0.001
T1	Ref				Ref			
T2	3.43	2.89	4.07	<0.001	3.27	2.74	3.90	<0.001
T3	10.23	8.87	11.80	<0.001	9.05	7.79	10.51	<0.001
T4	23.00	19.52	27.09	<0.001	19.40	16.31	23.10	<0.001

Abbreviations: NOS, not otherwise specified; Ref, reference; CI, confidence interval
